# Percutaneous Core Needle Biopsy Can Efficiently and Safely Diagnose Most Primary Bone Tumors

**DOI:** 10.3390/diagnostics11091552

**Published:** 2021-08-27

**Authors:** Vincent Crenn, Léonard Vezole, Amine Bouhamama, Alexandra Meurgey, Marie Karanian, Perrine Marec-Bérard, François Gouin, Gualter Vaz

**Affiliations:** 1 Orthopedics and Trauma Department, University Hospital Hotel-Dieu, CHU Nantes, 44000 Nantes, France; lvezole@gmail.com (L.V.); francois.gouin@unicancer.lyon.fr (F.G.); 2 Département de Chirurgie, Centre de Lutte Contre le Cancer Léon Bérard, 69008 Lyon, France; gualter.vaz@unicancer.lyon.fr; 3 PhyOs 1238, INSERM, Nantes University, UMR1238 Phy-Os “Bone Sarcomas and Remodeling of Calcified Tissues”, 44035 Nantes, France; 4 Département de Radiologie, Centre de Lutte Contre le Cancer Léon Bérard, 69008 Lyon, France; Amine.BOUHAMAMA@lyon.unicancer.fr; 5 Département d’anatomopathologie, Centre de Lutte Contre le Cancer Léon Bérard, 69008 Lyon, France; Alexandra.MEURGEY@lyon.unicancer.fr (A.M.); marie.karanian@lyon.unicancer.fr (M.K.); 6 Département d’oncologie Pédiatrique, Centre de Lutte Contre le Cancer Léon Bérard, 69008 Lyon, France; perrine.marec-berard@ihope.fr

**Keywords:** diagnostic accuracy, percutaneous biopsy, bone sarcoma

## Abstract

A biopsy is a prerequisite for the diagnosis and evaluation of musculoskeletal tumors. It is considered that surgical biopsy provides a more reliable diagnosis because it can obtain more tumor material for pathological analysis. However, it is often associated with a significant complication rate. Imaging-guided percutaneous core needle biopsy (PCNB) is now widely used as an alternative to surgical biopsy; it appears to be minimally invasive, possibly with lower complication rates. This study evaluates the diagnostic yield of the preferred use of PCNB in a referral center, its accuracy, and its complication rate. The data relating to the biopsy and the histological analysis were extracted from the database of a bone tumor reference center where PCNB of bone tumors was discussed as a first-line option. 196 bone tumors were biopsied percutaneously between 2016 and 2020. They were located in the axial skeleton in 21.4% (42) of cases, in the lower limb in 58.7% (115), and in the upper limb in 19.9% (39) cases. We obtained a diagnosis yield of 84.7% and a diagnosis accuracy of 91.7%. The overall complication rate of the percutaneous biopsies observed was 1.0% (*n* = 2), consisting of two hematomas. PCNB performed in a referral center is a safe, precise procedure, with a very low complication rate, and which avoids the need for first-line open surgical biopsy. The consultation between pathologist, radiologist, and clinician in an expert reference center makes this technique an effective choice as a first-line diagnosis tool.

## 1. Introduction

The incidence of primitive bone tumors is low, but they most commonly affect young adults. After a complete radiological examination, the great majority of bone tumors require a biopsy to determine whether they are benign or malignant, after which the appropriate therapeutic intervention can be determined. Surgical open biopsies are historically considered the gold standard in musculoskeletal tumor diagnosis as they offer the best diagnostic accuracy, around 98%, according to research [1,2]. Nevertheless, the performance of open biopsy comes with morbidity consequences [3], a high cost, and potential logistical difficulties that can delay its realization [4,5].

Recent studies have shown that percutaneous core needle biopsy (PCNB) results in a lower complication rate (0–2%) [3] than open biopsy (16%) [1,6]; it also results in shorter hospital stays and lower costs while maintaining high diagnostic accuracy. Its accuracy rates for bone tumors are not clearly defined and vary from 66 to 98% [4,6,7], with a higher diagnostic yield for bone lesions than for soft tissue lesions [8].

In this context, our hypothesis is that core needle biopsy carried out on primary bone tumors, when realized under optimal conditions in a musculoskeletal sarcoma reference center, offers a reliable diagnostic tool. This study evaluates the diagnostic yield and accuracy of first-line radio-guided PCNB in a referral center, as well as its complication rates.

## 2. Materials and Methods

### 2.1. Study Design

This retrospective monocentric study included all patients who underwent a diagnostic PCNB for a suspected bone tumor as initial care at the Centre Léon Bérard (CLB) referral musculoskeletal sarcoma center between 2016 and 2020. We assessed patient records for clinical context, tumor location, biopsy modalities and results, and complications following the procedure. This study was approved by the institutional research ethics committee. Cases of PCNB associated with radiological treatment (such as cryotherapy or ultra-sonotherapy) were excluded from this analysis. Solid metastatic tumor diagnoses were also excluded. A previous biopsy or prior diagnosis data were considered exclusion criteria as we focused on first-line diagnosis.

### 2.2. Biopsy Protocol

Percutaneous core needle biopsy of bone tumors was discussed as a first-line option in our therapeutic structure, and it was performed by experienced skeletal radiologists. Biopsy indication, choice between techniques, and anesthesia modalities were discussed in a weekly multidisciplinary team meeting. General anesthesia was preferred for young patients, or for complex and deep biopsy techniques requiring immobilization. When PCNB was validated, the needle tract was systematically approved after discussion between the interventional radiologist and the orthopedic surgeon.

**Figure 1:**
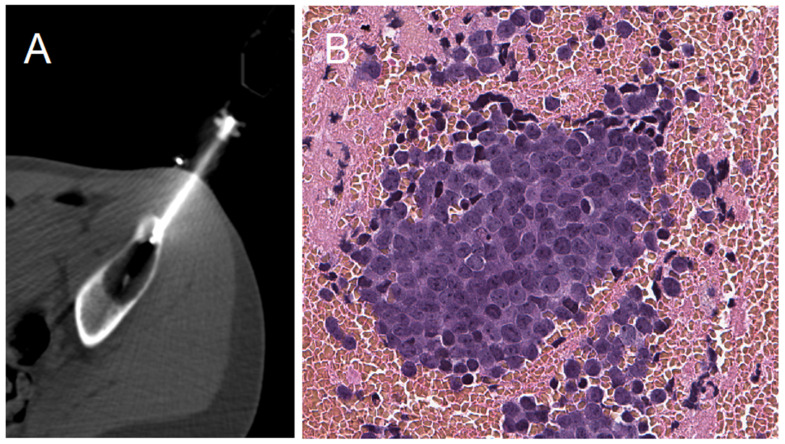
Percutaneous core needle biopsy CT-scan view and microscopic histological view. (**A**): PCNB CT-Scan axial view of a supra-acetabular iliac lytic aggressive lesion biopsy. (**B**): The final diagnosis of a Ewing sarcoma on a histopathological section, ×200 magnification.

Before each procedure, coagulation status was verified, and premedication was administered 30 min prior to the biopsy. The PCNBs were performed under CT-scan (Figure 1) (SOMATOM Emotion 16; Siemens Healthcare, Erlangen, Germany) or ultrasound (Aplio 500; Toshiba Medical Systems, Tokyo, Japan) guidance. For extra-osseous extension, a 13G coaxial needle biopsy (BARD Biopsy Systems, Crawley, UK) was inserted into the tumor and four to ten core needle biopsies were obtained using a 14G Tru-Cut biopsy needle (BARD Biopsy Systems, Crawley, UK). For intra-osseous lesions, we used an Arrow OnControl 13- or 11-gauge bone-access electric-powered device (Vidacare, Shavano Park, TX, USA). For osteoblastic lesions with no extra-osseous extension, we used an Arrow OnControl co-axial 12-gauge bone-access electric-powered device (Vidacare, Shavano Park, TX, USA), and two to three trephine biopsies were obtained using a 13G Arrow OnControl trephine. For osteolytic lesions, the same procedure was performed but four additional core needle biopsies were obtained using the 14G Tru-Cut biopsy needle. To allow further resection of the biopsy tract, the cutaneous needle entry point was tattooed with sterile China ink (Pigment RADSAFE noir; Biotic Phocea, Marseille, France) using MICROLET lancets (Bayer Healthcare, Neuilly-sur-Seine, France).

### 2.3. Outcome Measure

We evaluated the diagnostic yield, which was calculated in order to assess whether core needle biopsy can provide diagnostic information adequately. The pathology reports on all the biopsies were reviewed and categorized by pathologists as diagnostic and non-diagnostic specimens, or as insufficient specimens for pathological diagnosis. Patients who needed a second biopsy were also considered non-diagnostic. Patients who required an open biopsy or a second PCNB in order to obtain a final diagnosis were recorded. We determined the diagnostic accuracy after the first PCNB; accurate percutaneous biopsy results were considered to be those that were in agreement with the subsequent pathological examination results from the surgical specimens regarding the tumor’s histological nature (malignant or benign). Complications (i.e., hematoma, infection, fracture) in the month following biopsy were reported.

### 2.4. Statistics and Ethics

The data relating to the biopsy and the histological analysis were extracted from the database of a reference center for the management of musculoskeletal tumors where the PCNB of bone tumors was discussed as a first-line option. Data analyses were performed using SPSS in order to assess diagnostic parameters and associated results.

## 3. Results

### 3.1. Study Population

**Figure 2:**
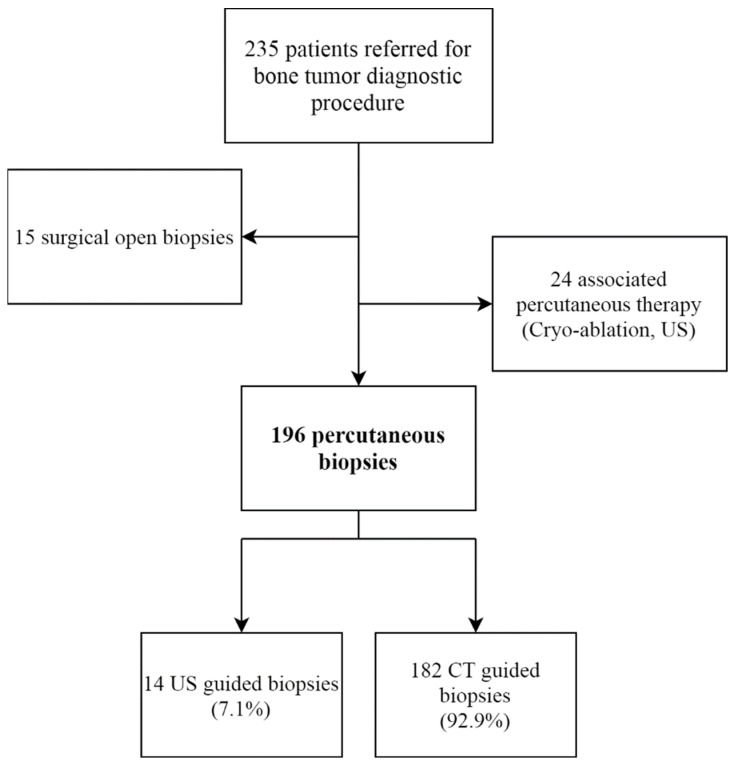
Study flow-chart. US: ultrasound; CT: computed tomography.

The study included 196 patients who underwent a core needle percutaneous diagnostic biopsy for a suspected bone tumor (Figure 2) (Table 1). Most PCNBs were performed under CT-Scan guidance (*n* = 182, 92.9%). Local anesthesia was used for 91.8% of patients (*n* = 180). For patients under 18 years old, local anesthesia was used for 75.0% (*n* = 9/12).

The final diagnosis revealed a malignant tumor in 49.5% of cases (*n* = 97), mostly osteosarcoma (13.8%), chondrosarcoma (13.8%), and Ewing sarcoma (10.2%). The most frequent benign lesion was a giant cell tumor (GCT) (*n* = 19; 9.7%) (Table 2).

### 3.2. Diagnostic Yield

**Figure 3:**
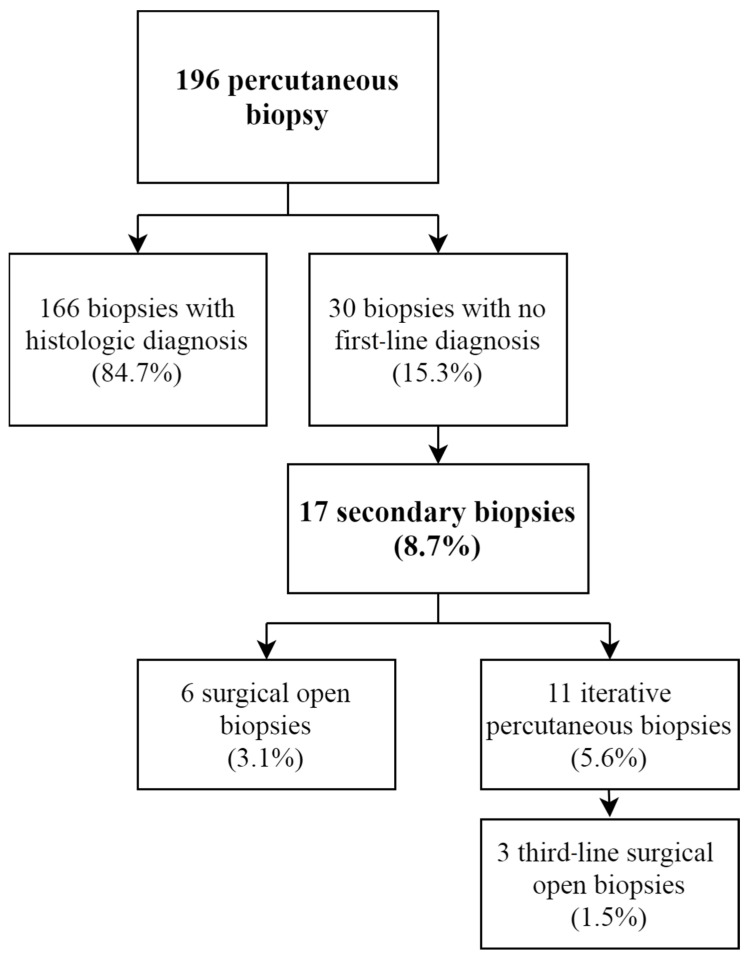
Percutaneous biopsy yield flow-chart.

The diagnostic yield after the first PCNB was obtained in 84.7% of cases, allowing a diagnosis and clear therapeutic strategy for 166 patients (Figure 3). For 30 patients (15.3%), no diagnostic certainty was observed; in two cases this was due to a technical failure to harvest sufficient tissue, and in the other cases (28 cases) the bone biopsy did not make it possible to obtain a final diagnostic certainty for diverse reasons (such as necrotic tissue, non-specific tissue or diagnostic doubt). Of these patients, 17 (8.7%) obtained a complementary biopsy, 6 of whom received an open surgical biopsy (3.1%); the remaining 11 received a second PCNB (5.6%). Other cases were considered benign lesions with no precise diagnosis (absence of malignant cells) after discussion in multidisciplinary team meetings, and were followed up radiographically. A third-line biopsy was performed surgically in an open fashion in three cases after an ineffective second-line PCNB.

### 3.3. Diagnostic Accuracy

We compared the first diagnosis proposed following PCNB with the definitive diagnosis obtained from the surgical specimen of 133 operated patients (67.8%). We obtained a PCNB accuracy of 91.7% (*n* = 122/133). Of the eleven remaining diagnoses with a discrepancy, eight were unable to obtain a formal initial diagnosis (72.7%). Second-line biopsies were performed for seven of the eleven diagnoses with a discrepancy (63.6%). Five of these were surgical open biopsies (45.4%) and two were CT-guided micro-biopsies (18.2%).

The second-line biopsies performed on these seven patients made it possible to obtain a final diagnosis in six cases, increasing the diagnostic accuracy to 96.2% (128/133).

**Figure 4:**
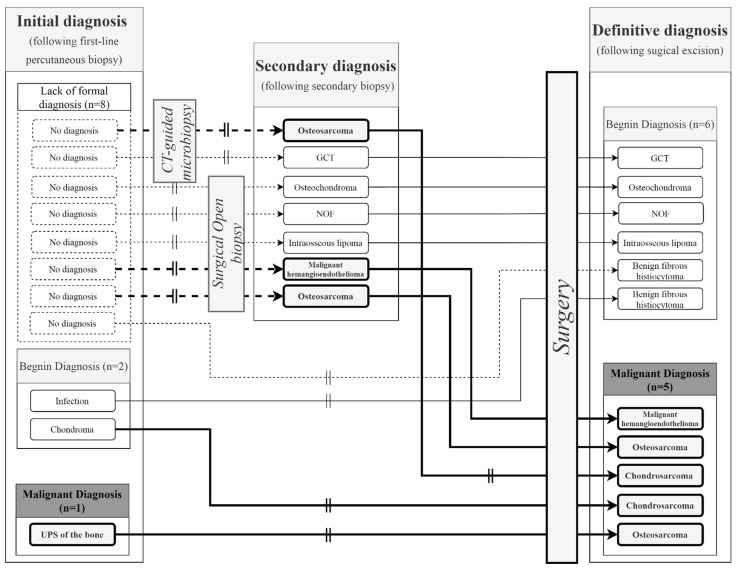
Focus on the 11 diagnostic discrepancies. The vertical double lines represent diagnostic discrepancy. The bold arrows represent patients with a final malignant diagnosis following a second biopsy (open or CT-guided) or direct surgery. The dotted arrows represent initial non-informative micro-biopsies with no diagnosis. GCT: Giant cell tumor; NOF: Non-ossifying fibroma.

Focusing on malignant lesion discrepancies, second-line biopsies made it possible to identify three malignant lesions (two osteosarcomas and one malignant hemangioendothelioma) (Figure 4). These were confirmed by surgical resection specimens, except for one chondroblastic osteosarcoma diagnosed with a second-line PCNB, which turned out to be a conventional chondrosarcoma, according to the final surgical diagnosis. One undifferentiated pleomorphic sarcoma of the bone on initial biopsy was an osteosarcoma, according to the surgical specimen. Finally, one initial diagnosis of chondroma was directly operated on after PCNB and was finally characterized as a chondrosarcoma, according to the surgical specimen. This was the only example of a discrepancy between benign to malignant, excluding secondary rectified diagnoses.

### 3.4. Complications

We observed a complication rate of 1.0%, consisting of only two hematomas, with no need for any treatment other than ice and medical surveillance. Neither infections nor fractures were observed among the cohort during the one-month follow-up.

## 4. Discussion

Until recently, open surgical biopsy was considered the gold standard for bone tumor diagnosis. Nonetheless, the inherent cost and iatrogenicity of open biopsy, balanced by considerable progress in histological characterization, are moving the cursor towards PCNB as a first-line diagnosis tool for skeletal tumors, with evolving reference guidelines [9]. Our cohort confirmed this recent trend, as we obtained a high diagnostic accuracy of 91.7%, associated with an 84.7% diagnostic yield. Moreover, no complications, apart from two cases of hematoma (1.0%), were observed using the PCNB technique.

### 4.1. Diagnostic Yield

In our experience, this technique yielded sufficient amounts of tissue to make a diagnosis in 84.7% of cases, with the need for a second biopsy in 8.7% of cases. The diagnostic yield seems acceptable, and a secondary PCNB might be easily proposed in the case of a non-contributive biopsy (*n* = 11, 5.6%) or a lack of certainty. Complementary surgical open biopsy was necessary in nine cases (4.6%), as a second-line (*n* = 6, 3.1%) or third-line (*n* = 3, 1.5%) diagnostic tool. Most of the uncertain diagnoses consisted of benign lesions with no need for surgery and were simply followed up radiologically; this consideration is corroborated by the high diagnostic accuracy observed for bone tumors requiring a surgical excision strategy. These findings are in line with the conclusions in the meta-analysis by Kubo et al., which suggest that a first-line core needle biopsy should be performed by expert radiologists, and that a surgical biopsy should be performed secondarily if diagnosis following the core needle biopsy does not match the clinical presentation and radiographic findings [10].

### 4.2. Diagnostic Accuracy

The accuracy of PCNB for operated patients with a definitive histological diagnosis from a surgical specimen is a methodologically high-value criterion. For this specific outcome, we obtained a 91.7% diagnostic accuracy. Moreover, six (54.5%) of the initial no-diagnoses were completed successfully with a second-line biopsy, increasing diagnostic accuracy to 96.2%. These results are consistent with recent research on this subject: in bone and soft tissue tumors, Yang et al. achieved a diagnostic accuracy of 89% in 509 cases [11]; Klein et al. observed 83.6% in their study [12].

It is also of interest to consider that two of the five malignant discrepancies (40.0%) were due to a cartilaginous tumor, which presents well-known difficulties in bone tumor diagnosis. In fact, for this specific histological type, it was possible to confirm only the chondral nature of the lesion using PCNB and not its aggressiveness, as bone resorption images are absent on these samples most of the time [9]. Chondral tumors specifically require a multidisciplinary approach for accurate diagnosis, associating multimodal imaging analysis, as stated by Sharif et al. [13]. Moreover, these data should also be correlated with the clinical presentation, and it is only under these circumstances that the tumor board can propose a therapeutic strategy [14].

We observed only one event (0.8%) with a discrepancy in which the diagnosis went from benign to malignant, excluding rectified diagnoses on second biopsy. Within this criterion, our findings are in line with previous studies confirming the reliability of PCNB for differentiating between benign and malignant lesions, even in aggressive radiolucent bone tumors [15,16].

### 4.3. Complications

One of the major points of interest regarding the use of PCNB in bone tumor diagnosis remains its low rate of complications. Our cohort confirmed this tendency with a rate of complications of one per cent (1.0%, *n* = 2), consisting only of two hematomas, with no need for surgical reoperation. This exceptionally low rate compared to that of open biopsy, which is up to 16% [17,18], is of great interest, as an infection or a fracture following a biopsy can drastically worsen the prognosis for bone sarcoma.

### 4.4. Limitations and Strengths

Some of the patients in the cohort were not included in the study, such as those who had a direct open biopsy or an associated therapeutic treatment. Moreover, as a consequence of the retrospective design, it was not always possible to precisely obtain the decision process that guided the second-line biopsy towards an open biopsy or PCNB.

As proposed by some authors [4], we also decided to not consider in the precision parameter calculation any patients with no surgical confirmation of their diagnosis, as our analyses were based on histological accuracy. We decided that it would be inaccurate to confirm or deny a precise histological diagnosis based only on patient follow-up.

This work, carried out in a specialized sarcoma center, is of interest because of its cohort size and homogeneity, focusing especially on bone tumors. In this context, it seems important to remember that a key factor for external validation and the appropriate use of PCNB is an experienced musculoskeletal tumor team that communicates frequently and is capable of correlating clinical, radiographic, and histological information for each patient.

## 5. Conclusions

Percutaneous core needle biopsy with radio-guided drilling performed in a referral center is a safe, precise procedure, with a low complication rate, that prevents the need for a first-line open surgical biopsy. Even though its yield is lower than that of open biopsy, its accuracy concerning operated lesions remains at a high level, at 91.7%. Moreover, a second-line biopsy in cases with a lack of certainty raises the diagnostic accuracy to 96.2%. Concerted work with anatomo and pathology teams in expert reference centers makes this technique an effective choice as a first-line diagnostic tool.

## Figures and Tables

**Table 1:** Study population data.

	Study Cohort (*n* = 196)
Age	41.9 (8–90)
Sex	
Female	106 (54.1%)
Male	90 (45.9%)
Performance status	
0	154 (78.6%)
1–2	37 (18.9%)
3–4	5 (2.5%)
Localization	
Upper limb	39 (19.9%)
Lower limb	115 (58.7%)
Axial skeleton	42 (21.4%)
Biopsy type	
Percutaneous guided	
Echography	14 (7.1%)
CT	182 (92.9%)
Anesthesiology modality	
Local anesthesia	180 (91.8%)
General anesthesia	16 (8.2%)

**Table 2:** Final histopathology results for bone tumors. *: no specific diagnosis, but no atypical cells nor malignancy detected.

Sampling Outcome Diagnosis		Final Diagnosis	
Malignant		Begnin	
Osteosarcoma	27 (13.8%)	Giant cell tumor	19 (9.7%)
Chondrosarcoma	27 (13.8%)	No malignancy lesion *	18 (9.2%)
Ewing sarcoma	20 (10.2%)	Angioma	7 (3.6%)
Lymphoma	11 (5.6%)	Chondroblastoma	7 (3.6%)
Chordoma	4 (2.0%)	Osteoid Osteoma	5 (2.6%)
Myeloma or plasmacytoma	4 (2.0%)	Nonossifying fibroma	5 (2.6%)
High-grade sarcoma (NOS)	1 (0.5%)	Langerhans cell histiocytosis	5 (2.6%)
Pleomorphic cell sarcoma	1 (0.5%)	Osteochondroma or osteoma	4 (2.0%)
Adamantinoma	1 (0.5%)	Fibrous dysplasia	4 (2.0%)
Malignant hemangioendothelioma	1 (0.5%)	Infection	3 (1.5%)
		Simple bone cyst	3 (1.5%)
		Benign fibrous histiocytoma	3 (1.5%)
		Chondroma	2 (1.0%)
		Osteoblastoma	2 (1.0%)
		Reactive bone changes	2 (1.0%)
		Paget’s disease	2 (1.0%)
		Begnin hemangioma	2 (1.0%)
		Atypic cartilaginous tumor	1 (0.5%)
		Brown tumor	1 (0.5%)
		Intra-osseous lipoma	1 (0.5%)
		Gout tophus	1 (0.5%)
		Chondrocalcinosis	1 (0.5%)
Total	97(49.5%)		99 (51.5%)

## Data Availability

The datasets generated and/or analyzed during this study are available from the corresponding author on reasonable request.
